# The Spread of SARS-CoV-2 Omicron Variant in CALABRIA: A Spatio-Temporal Report of Viral Genome Evolution

**DOI:** 10.3390/v15020408

**Published:** 2023-01-31

**Authors:** Claudia Veneziano, Nadia Marascio, Carmela De Marco, Barbara Quaresima, Flavia Biamonte, Enrico Maria Trecarichi, Gianluca Santamaria, Angela Quirino, Daniele Torella, Aldo Quattrone, Giovanni Matera, Carlo Torti, Caterina De Filippo, Francesco Saverio Costanzo, Giuseppe Viglietto

**Affiliations:** 1 Department of Experimental and Clinical Medicine, “Magna Graecia” University of Catanzaro, 88100 Catanzaro, Italy; 2 Interdepartmental Center of Services (CIS), Molecular Genomics and Pathology, “Magna Græcia” University of Catanzaro, 88100 Catanzaro, Italy; 3 Department of Health Sciences, “Magna Graecia” University of Catanzaro, 88100 Catanzaro, Italy; 4 Department of Medical and Surgical Sciences, “Magna Graecia” University of Catanzaro, 88100 Catanzaro, Italy; 5 “Mater Domini” University Hospital of Catanzaro, 88100 Catanzaro, Italy; 6 Neuroscience Research Center, “Magna Graecia” University of Catanzaro, 88100 Catanzaro, Italy

**Keywords:** SARS-CoV-2, variants, surveillance, NGS, viral genomics, Italy

## Abstract

We investigated the evolution of SARS-CoV-2 spread in Calabria, Southern Italy, in 2022. A total of 272 RNA isolates from nasopharyngeal swabs of individuals infected with SARS-CoV-2 were sequenced by whole genome sequencing (N = 172) and/or Sanger sequencing (N = 100). Analysis of diffusion of Omicron variants in Calabria revealed the prevalence of 10 different sub-lineages (recombinant BA.1/BA.2, BA.1, BA.1.1, BA.2, BA.2.9, BA.2.10, BA.2.12.1, BA.4, BA.5, BE.1). We observed that Omicron spread in Calabria presented a similar trend as in Italy, with some notable exceptions: BA.1 disappeared in April in Calabria but not in the rest of Italy; recombinant BA.1/BA.2 showed higher frequency in Calabria (13%) than in the rest of Italy (0.02%); BA.2.9, BA.4 and BA.5 emerged in Calabria later than in other Italian regions. In addition, Calabria Omicron presented 16 non-canonical mutations in the S protein and 151 non-canonical mutations in non-structural proteins. Most non-canonical mutations in the S protein occurred mainly in BA.5 whereas non-canonical mutations in non-structural or accessory proteins (ORF1ab, ORF3a, ORF8 and N) were identified in BA.2 and BA.5 sub-lineages. In conclusion, the data reported here underscore the importance of monitoring the entire SARS-CoV-2 genome.

## 1. Introduction

In December 2019, a novel coronavirus, severe acute respiratory syndrome coronavirus 2 (SARS-CoV-2) [1] caused an outbreak of atypical pneumonia in Wuhan, China [2], designated by the World Health Organization (WHO) as Coronavirus Disease 2019 (COVID -19). The Wuhan strain of SARS-CoV-2 (NC_045512.2) was rapidly replaced in late January 2020 by a variant called B.1, characterized by mutation D614G in the spike protein (S protein) gene, which increases the transmissibility of SARS-CoV-2 [3]. The B.1 lineage spread worldwide in March 2020 [4]. The rapid spread of SARS-CoV-2 was triggered by the increase in mutations in the S protein that enhanced binding to the ACE2 receptor [5], counteracted the neutralizing effect of natural antibodies [6,7], and/or increase the transmissibility of the virus to other species [8,9,10,11]. In November 2020, the variant of concern (VOC) designated Alpha (B.1.1.7, according to Pango nomenclature [12]) emerged in the United Kingdom. The Alpha variant had nine mutations in the S protein, including the N501Y mutation that promotes infection and transmission of SARS-CoV-2 [13]. In late 2020, two additional VOCs named Beta (B.1.351, according to Pango nomenclature [12]) and Gamma (P.1, according to Pango nomenclature [12]) emerged in South Africa and Brazil, respectively. The beta variant had nine mutations in the S protein, including mutations K417N and E484K, which play an important role in bypassing monoclonal antibodies and increasing transmissibility [14]. The Gamma variant was also characterized by higher transmissibility and by the ability to evade the immune system [15]. The Gamma variant presented 17 mutations of the S protein, including H655Y, K417N, and E484K [16]. In May 2021, the Delta variant (B.1.617.2, according to Pango nomenclature [12]) emerged in India. Compared to the previous VOCs, the Delta variant was found to be even more transmissible [17] and infectious [18] due to mutations L452R, T478K, and P681R. The Omicron variant (B.1.1.529, according to the Pango nomenclature [12]) SARS-CoV-2 occurred in South Africa and Botswana on 24 November 2021 [19]. Despite the high immunity of the global population, the Omicron variant rapidly spread around the world, triggering new COVID-19 outbreaks in late 2021 [20,21,22]. The Weekly Epidemiological Update published by WHO (11 January 2022), showed that in the first 7 days of 2022, the number of new cases increased by 100% worldwide. After the discovery of the first case of an Omicron patient in Italy in early December 2021, the Italian Integrated Surveillance System found that the Omicron variant became dominant in Italy in less than a month [23]. In early 2022, mutations that led to the emergence of Omicron were used to define dozens of phylogenetical sub-lineages, including BA.2, BA.3, BA.4, BA.5 [24,25], and many others that are continuously emerging worldwide. Compared to other VOCs, Omicron sub-lineages are more easily transmissible and escape neutralizing antibodies produced by previous infections or vaccinations, resulting in an increased number of infections and/or breakthroughs [22,26,27,28]. Omicron represents the variant that has accumulated the greatest number of missense mutations along the viral genome (range, 49–57) compared to all the previous VOCs [29,30]. Overall, the Omicron genome was affected by mutations in a minimum of six viral genes (such as in the BA.1 sub-lineage) to a maximum of nine viral genes (such as in the BA.4 sub-lineage). The highest number of mutations in the Omicron genome was observed in the gene encoding the S protein (range, 29–34). Many of the mutations observed in the gene encoding the S protein of the Omicron variant were common to previous VOCs [31,32]. For example, del69/70 appeared for the first time in the Alpha variant and is present in Omicron sub-lineages BA.1, BA.4, and BA.5; N501Y is typical of Alpha, Beta, Gamma, and of all Omicron sub-lineages; L452R, a characteristic mutation of Delta, reappeared in Omicron sub-lineages BA.4 and BA.5. Notably, several mutations present in viral proteins different from S (ORF1ab, ORF3a, M, ORF6, ORF7a/b, N) of Omicron are conserved. For example, deletion del3675/3677 in ORF1ab has evolved in Alpha, Beta, Gamma, and Omicron whereas the combination of R203K and G204R in the N protein is typical of Alpha, Gamma, and Omicron. As a consequence of the emergence of Omicron sub-lineages, the European Medicines Agency (EMA) recently approved the administration of an adapted version of the mRNA vaccine COVID-19 Comirnaty (Pfizer/BioNTech) for Omicron sub-lineages BA.4 and BA.5 [33] and WHO updated public health surveillance guidelines for COVID-19 [19], which includes monitoring the circulation of VOCs by genomic surveillance. In the present study, we report on the monitoring of circulating Omicron sub-lineages during 2022 in Calabria, Southern Italy.

## 2. Materials and Methods

### 2.1. Patients

All the data used in this study were previously anonymized as required by the Italian Data Protection Code (Legislative Decree 196/2003) and the general authorizations issued by the Data Protection Authority. The project was approved by the Ethical Committee of Regione Calabria in the Meeting no. 434 held on 16 December 2021. We collected nasopharyngeal swab specimens from 272 SARS-CoV-2-positive patients in 4 Calabria areas: Catanzaro, Vibo Valentia, Crotone, and Reggio Calabria. RNAs from 172 swabs were subjected to whole genome sequencing (WGS), while RNAs from 100 patients were sequenced by the Sanger method. The complete list of patients with demographic information and sequencing methods is reported in Table S1.

### 2.2. Sequencing of Viral Genome

The SARS-CoV-2 nasopharyngeal positive swabs were collected in the hospitals of the Catanzaro area, Southern Calabria, Italy. The diagnosis was carried out by the following assays: Allplex SARS-CoV-2 Variants (Arrow Diagnostics Srl, Genoa, Italy), Applied Biosystems TaqPath COVID-19 high throughput combo (ThermoFisher Scientific, Waltham, MA, USA), Xpert^®^ Xpress SARS-CoV-2 (Cepheid, Sunnyvale, CA, USA) and/or cobas^®^ SARS-CoV-2 Test (Roche Diagnostics, Indianapolis, IN, USA). Viral RNA was extracted using the NUCLISENS^®^ easyMAG^®^ (bioMérieux, Florence, Italy). The RNA samples were subjected to retro-transcription through the InvitrogenTM SuperScript IV VILO Master Mix (ThermoFisher Scientific) and then S gene regions were amplified with the primer list reported in Table S2 and using PCR products obtained through the BigDye Direct Cycle Sequencing Kit (Applied Biosystems, Waltham, MA, USA) were then purified according to the instructions of the BigDye Terminator Purification Kit (Applied Biosystems) and sequenced through the Genetic Analyzer 3500 Dx (Applied Biosystems). Electropherograms were analyzed by SeqScape Software, v.4 (Applied Biosystems), and compared to the SARS-CoV-2 reference genome (NC_045512.2). In brief, 7 μL of viral RNA were retrotranscribed by using InvitrogenTM SuperScriptTM VILO cDNA Synthesis Kit (ThermoFisher Scientific). Libraries were prepared using the Ion AmpliSeq SARS-CoV-2 Research Panel (ThermoFisher Scientific), which consists of 237 amplicons ranging from 125 to 275 bp in length across the SARS-CoV-2 genome. Libraries preparation was performed either manually according to the Ion AmpliSeq Library Kit Plus or on the automatic Ion ChefTM Instrument by using the Ion AmpliSeq Kit for Chef DL8 (ThermoFisher Scientific). The final concentration of manually prepared cDNA libraries was determined on the Agilent 2200 System by the Agilent High Sensitivity DNA Assay (Agilent Technologies, Santa Clara, CA, USA), following manufacturer’s recommendations. The concentration of automatically prepared cDNA libraries was determined by Ion Library TaqMan Quantitation Kit (ThermoFisher Scientific). Barcoded libraries were diluted to 30 pM and then loaded onto the Ion ChefTM Instrument (ThermoFisher Scientific) for emulsion PCR, enrichment, and loading onto the Ion S5 520 chip. Post-sequencing run analysis was performed by the Ion Torrent Suite Software with the following plugins: The SARS-CoV- 2_coverageAnalysis, COVID19AnnotateSnpEff for variant annotation, and generateConsesus to obtain consensus sequence for each barcode. Variant classification was performed on whole-genome sequences using the Pangolin tool 4.1.1 [34].

### 2.3. Phylogenetic Analysis

Phylogenetic analysis was performed using the full FASTA dataset via NGPhylogeny web tool [35] on FastTree [36] default parameters (bootstrap = 100). Visualization of the phylogenetic tree was performed by Interactive Tree of Life (iTOL) v6 software [37].

### 2.4. Analysis of Viral Strains

Distribution of Omicron sub-lineages in Italy was extracted from GISAID database (data extraction 30 September 2022) using the filters human, VOC Omicron GRA, Italy, complete and low coverage excluded. A total of 48,453 sequences were extracted (Table S3). The frequency of non-canonical mutations in S protein in the viral isolates in Italy was extracted from GISAID database using the filters human, VOC Omicron GRA, complete, low coverage excluded (Table S3).

## 3. Results

### 3.1. Evolution of Omicron Sub-Lineages

**Figure 1:**
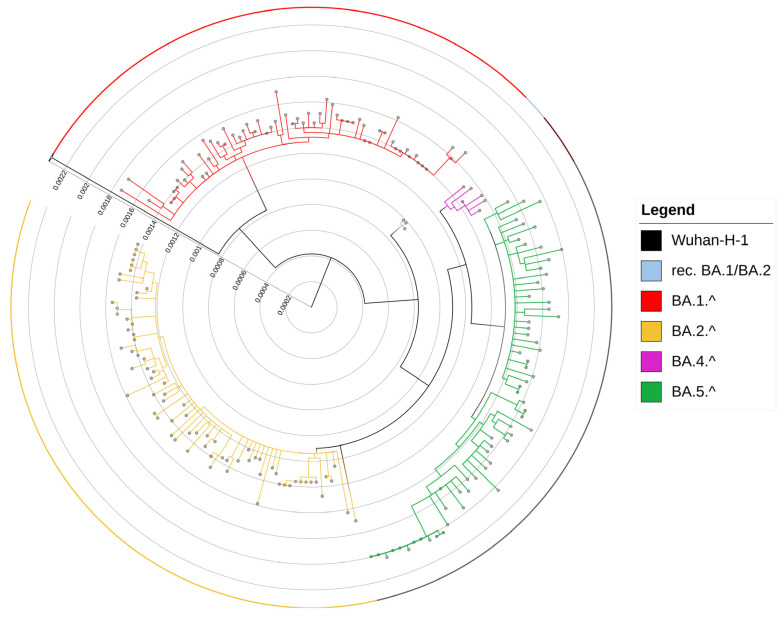
Omicron lineages isolated in Calabria during 2022: phylogenetic analysis. The phylogenetic tree shows the genetic relationship between the 208 Omicron isolates included in the study. Bootstrap equal to 100 was used. The genetic distance is reported within the graph.

The total number of COVID-19 patients in the Calabria region in 2022 was 324,138 [38] distributed as follows: 18,935 in January, 24,347 in February, 51,961 in March, 50,305 in April, 38,756 in May, 21,281 in June, 59,969 in July, 35,410 in August and 23,174 in September (Table S4). From January 2022 the highest number of infections was registered in the Reggio Calabria province (N = 135,090 cases), followed by Catanzaro province (N = 68,421 cases), Crotone province (N = 44,896 cases) and Vibo Valentia province (N = 34,227 cases). The percentage of positive swabs per month in the specific regional area was on average 44% (range 22–85%) for Reggio Calabria, 22% (range 8–28%) for Catanzaro, 14% (range 8–25%) for Crotone and 11% (range 6–24%) for Vibo Valentia, (Table S4). The percentage of sequenced samples compared to positive swabs in the Calabria area was on average 0.4% (range 0.1–0.8%). As previously reported [39], Calabria Omicron arose in January 2022 and became the prevalent variant in February 2022. To investigate the evolution of the Omicron variant in Calabria, in this study we have analyzed SARS-CoV-2 swabs obtained during 2022, including previously published data that referred to January and February 2022. Viral RNA was extracted from 272 nasopharyngeal swabs that were positive between March and September 2022, of which 172 were subjected to whole genome sequencing while 100 were sequenced by targeted Sanger sequencing of specific regions of S, M, and N genes (see Section 2). As previously indicated SARS-CoV-2 NGS sequences relative to January and February 2022 were already published [39] and available in the GenBank [40] under accession No. SUB11466277. As to the swabs sequenced in this study, the median depth of sequencing was 4200 (range, 790–11,169), with genome coverage > 99% in 97% of the viral genome. A median number of 613,908 reads (range 110,033–1,618,768) was generated for each sample (Table S5). For data relative to sequences of swabs collected in January and February 2022, see De Marco et al., 2022 39. From January 2022, Omicron was the prevalent variant in the Calabria region. Phylogenetic tree analysis of the complete genomes of Omicron isolates is shown in Figure 1. 

**Figure 2:**
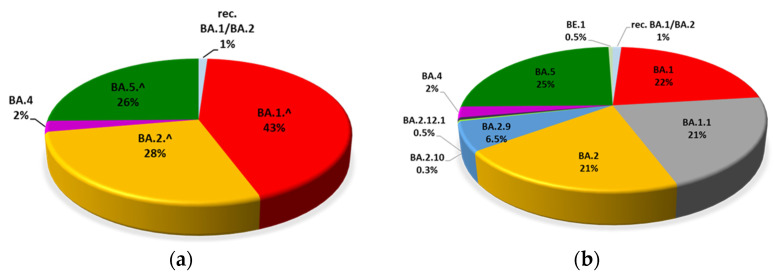
Frequency of Omicron lineages (**a**) and sub-lineages (**b**) in the cohort under analysis observed in Calabria during 2022 (legend: rec, recombinant).

Through this analysis, we have identified five Omicron lineages that were further divided into different sub-lineages. The five Omicron lineages identified in this study were: recombinant BA.1/BA.2, BA.1.^ (sub-lineages BA.1, BA.1.1), BA.2.^ (sub-lineages BA.2, BA.2.9, BA.2.10, BA.2.12.1,), BA.4.^ (sub-lineage BA.4), BA.5.^ (sub-lineages BA.5, BE.1) (Figure 2a). In total, from January 2022 we have identified four recombinant BA.1/BA.2 isolates (1%), 81 BA.1 (22%), 78 BA.1.1 (21%), 76 BA.2 (21%), 24 BA.2.9 (6.5%), 1 BA.2.10 (0.3%), 2 BA.2.12.1 (0.5%), 9 BA.4 (2%), 91 BA.5 (25%) and 2 BE.1 (0.5%) (Figure 2b).

**Figure 3:**
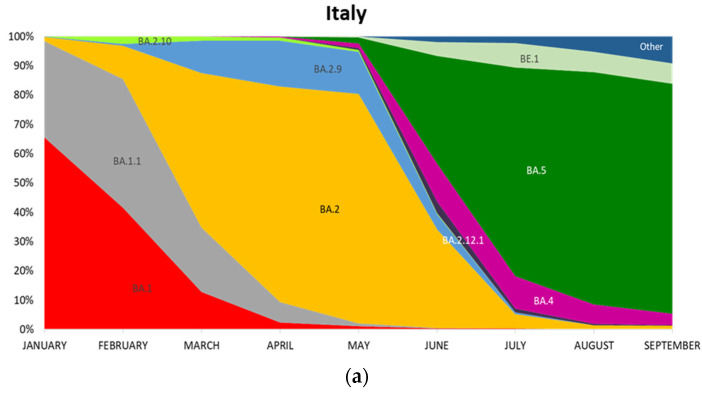

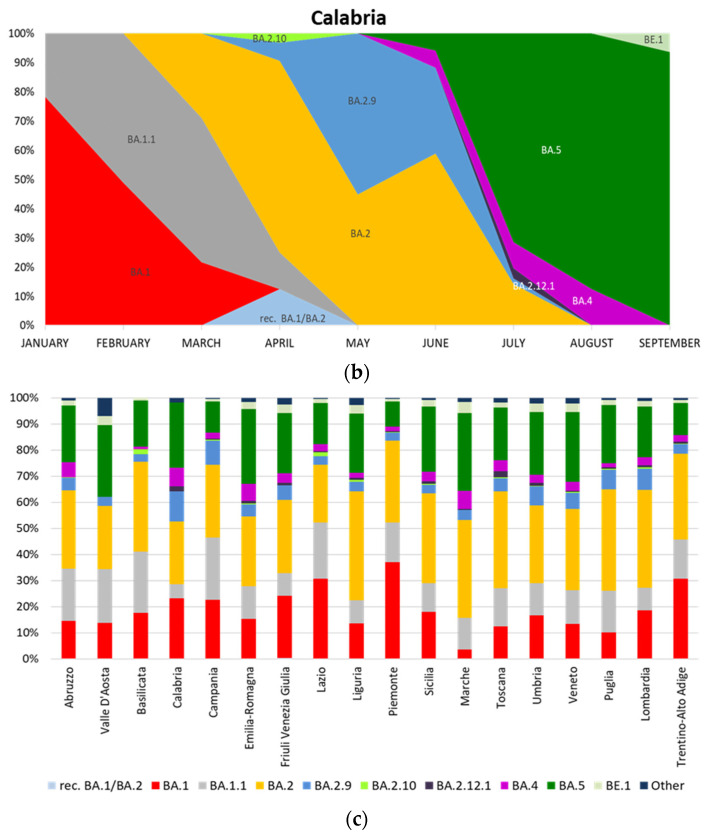
Distribution of Omicron sub-lineages during 2022. (**a**) Monthly distribution of Omicron sub-lineages in Italy during 2022 (N = 48,453). (**b**) Monthly distribution of Omicron sub-lineages in Calabria during 2022 (N = 368). (**c**) Omicron sub-lineages distribution in Italian regions. Data were extracted from the GISAID database on 30 September 2022 (legend: rec, recombinant).

Subsequently, we compared the distribution of the 10 Omicron sub-lineages in Italy (data extracted from the GISAID database) (Figure 3a) with the prevalence of Omicron sub-lineages present in Calabria (Figure 3b) as observed in this study (Table 1). The comparison of Omicron spread in Calabria and Italy showed a similar trend as to the emergence of Omicron sub-lineages, with few exceptions. BA.1 showed persistence in Italy mainly during April and May different from what occurred in Calabria where BA.1 disappeared in April. Recombinant BA.1/BA.2 showed increased frequency in Calabria in April (13%) compared to Italy, where its frequency was negligible (0.02% between April and June). BA.2.9 emerged in Italy already in February whereas this sub-lineage was detected in Calabria for the first time in April. Finally, BA.4 and BA.5 spread to Italy in May whereas the first cases in Calabria were detected in June. The distribution of the different sub-lineages in the four Calabria areas (Catanzaro, Vibo Valentia, Crotone, Reggio Calabria) is shown in Figure S1 and Table S4. The distribution of the 10 Omicron sub-lineages present in the GISAID database (on 30 September 2022) showed, as a whole, a similar behavior throughout all Italian regions, though with some notable exceptions. The highest frequency of BA.1 in Italy occurred in Lazio, Piemonte, and Trentino-Alto Adige (31%, 37%, and 31%, respectively), whereas the lowest frequency was observed in Marche (4%). Conversely, Calabria was the Italian region with the highest frequency of BA.2.9 variant (12%). As described, BA.2.9 appeared in Calabria in April 2022 and remained until July 2022 with a frequency higher than that observed in Italy (1%). In addition, from the analysis of the Gisaid database Calabria, Emilia-Romagna, and Marche showed a frequency of 7% for BA.4, which is the highest frequency of this sub-lineage observed in Italy during 2022 (Figure 3c).

In this study, mutations that allowed the identification of a specific VOC were indicated as canonical, whereas mutations that appeared sporadically in one or more isolates were indicated as non-canonical. Both canonical and non-canonical mutations were present in the S gene, in the genes encoding both structural (E, M, and N) and non-structural viral proteins (ORF1ab, ORF3a, ORF6, ORF7a, ORF8) [41].

### 3.2. Mutations in the S Protein

Omicron lineages (recombinant BA.1/BA.2, BA.1.^, BA.2.^, BA.4.^, BA.5.^) were characterized by 29–34 canonical mutations in the gene encoding S protein as listed in Table S6 [41]. The first Omicron lineage that appeared in South Africa was BA.1.^, a variant that presents 34 canonical mutations in the S protein. BA.1.^ also presented the H69/V70 deletion that initially characterized the Alpha variant. H69/V70del was conserved in BA.4.^ and BA.5.^ lineages [41]. Interestingly, two additional mutations in the S protein that were characteristic of the Alpha variant, N501Y, and P681H, but not in the Delta variant, reappeared in all Omicron lineages [41]. Similarly, all Omicron lineages showed the canonical mutation H655Y that appeared for the first time in Gamma. 

Recombinant BA.1/BA.2 described in this study shared all canonical mutations of S protein (N = 29) with BA.2.^ lineage. 

Among the 29 canonical mutations, one (deletion P25/A27del) was present also in lineages BA.4.^ and BA.5.^. BA.4.^ and BA.5.^ shared 31 canonical mutations in S protein, including L452R, a mutation that appeared for the first time during the wave of Delta variant. Additional canonical mutations in the S protein common to all Omicron lineages, identified in this study, were G339D, S371F, S373P, S375F, K417N, N440K, S477N, T478K, Q498R, Y505H, N679K, Q954H, N969K. 

Notably, canonical mutations of the S protein prevalently occurred in the Receptor Binding Domain (RBD). The mutations in the RBD domain were distributed as follows: 16 canonical mutations in recombinant BA.1/BA.2, BA.1.^ and BA.2.^; 17 in BA.4.^ and BA.5.^. 

Concerning other domains, the N-terminal domain (NTD) was characterized by five mutations in recombinant BA.1/BA.2 and BA.2.^, eight in BA.1.^; 6 in BA.4.^ and BA.5.^; C-terminal 1– C-terminal 2 domain (CT1/CT2) showed four canonical mutations in all lineages; S1/S2 cleavage site domain presented two canonical mutations in all lineages; Hepta repeat 1 (HR1) domain showed two canonical mutations in recombinant BA.1/BA.2, BA.2.^, BA.4.^ and BA.5.^ and 3 in BA.1.^. Notably, only lineage BA.1.^ showed N856K in the Fusion peptide (FP).

We have also observed that the S protein of Omicron lineages presented 16 non-canonical mutations (L5F, V6I, P9T, S12F, T22I, M153I, D178N, G181V/A, P209L, I624M, N658S, A701V, A713S, P862S, E1144V). The most frequent non-canonical mutations in the cohort under analysis were L5F and A701V (2.5% and 2.7%, respectively), with N658S and A713S showing lower frequencies (0.8% and 0.5%, respectively). All the remaining non-canonical mutations (V6I, P9T, S12F, T22I, M153I, D178N, G181V/A, P209L, I624M, P862S, E1144V) appeared in only one isolate. L5F was the only non-canonical mutation that appeared in two lineages (BA.2.^, and BA.5.^). 

**Figure 4:**
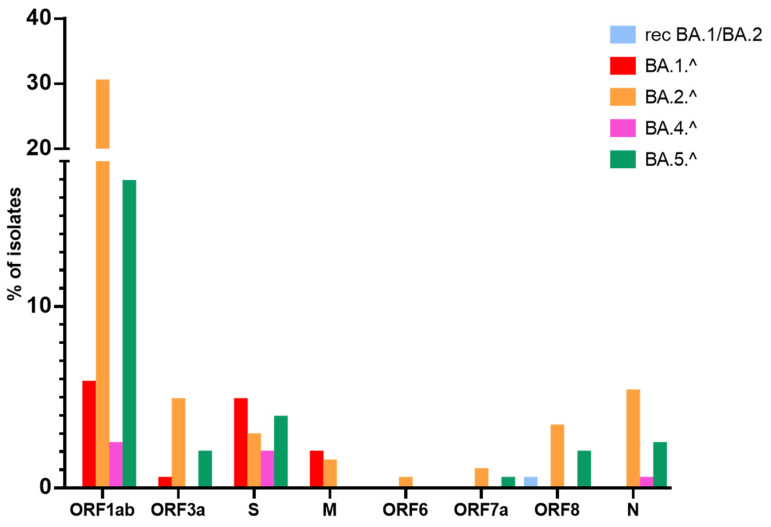
Percentage of isolates with non-canonical mutations in the different SARS-CoV-2 genes. Histograms show the percentage of isolates belonging to a particular lineage that have non-canonical mutations in the indicated viral genes. (Legend: ORF. Open Reading Frame; S. Spike; M. membrane; N. nucleocapsid, rec. recombinant).

Notably, the lineages that presented the highest percentage of isolates with non-canonical mutations were BA.1^ (4.8%), BA.5.^ (3.9%), and BA.2.^ (2.9%). Concerning the lineages with a greater number of non-canonical mutations, BA.5.^ showed nine different non-canonical mutations (L5F, S12F, M153I, G181V/A, P209L, A713S, P862S, E1144V). In total, 36 isolates (10%) had only 1 non-canonical mutation in the S protein. Details are reported in Table 2 and Figure 4.

Concerning the timing of the emergence of non-canonical mutations, we have observed that A701V was first detected in nine patients in January, P9T and T22I appeared each in one patient in March, L5F was detected in three patients in April, I624M was detected in one patient in May, V6I, M153I, N658S were detected in five patients in June, S12F, D178N, G181A, P209L, and A713S were detected in six patients in July, G181V and P862S were detected in one patient in August, and finally, E1144V was detected in one patient in September. Notably, the highest frequency of occurrence of non-canonical mutations in the S protein was observed in July (31%). Interestingly, most non-canonical mutations were in the NTD, CT1-CT2, S1/S2 cleavage site, and FP domains of the S protein [42] but not in the RBD domain (Table 3).

Some mutations are apparently of particular interest since they are located within (M153I) or near (P9T, S12F and T22I) the antigenic supersite of the NTD domain, which represents the main target of antibodies produced by memory B cells after administration of mRNA vaccine [43,44]. Three additional non-canonical mutations (N658S, A701V, A713S) were located between CT1/CT2 and S1/S2 cleavage site and may be involved in the increase in cleavage capability of the S protein [16]. Finally, N658S, identified in three BA.4 isolates, is implicated in resistance to antibody neutralization [45]. 

P862S is a mutation that falls in the FP domain. It showed a frequency of 0.3% in the cohort under analysis. Notably, during the Omicron wave, P862S mutation occurred with a very low frequency in Europe (0.0008%) whereas it was detected in a single patient from the Italian region, Trentino-Alto Adige, in April 2022 (GISAID database). This may be due to the observation that residue P862 in the FP domain may impair viral entry because it is necessary to initiate viral fusion with the host cell membrane [46,47]. Accordingly, only a small number of mutations in the FP domain have been described during the pandemic, possibly because this domain is highly conserved among β-coronaviruses [48,49]. The only mutation in this domain described worldwide is N856K with an overall frequency of 43% [41]. 

The mutation density per domain defined as the number of mutations divided by the amino acidic size of the domain was calculated and reported in Table 3. The highest mutation density was shown by the NTD domain (3.4%) followed by the CT1-CT2 (1.3%), S1/S2 cleavage site (1.6%), and FP (1.1%) domains (Table 3). Among the different Omicron lineages, BA.5.^ and BA.2.^ presented the highest number of mutations in the NTD domain (N = 7 and N = 2, respectively), compared to BA.1.^ and BA.4.^ (N = 1). It is of note that BA.5.^ also showed the major number of mutated domains compared to other lineages (N = 4). See Table 3.

### 3.3. Mutations in Structural Proteins

Structural proteins E, M, and N play crucial roles in viral replication, assembly, and release [50]. Mutations identified so far in the above-mentioned proteins are listed in Table S7 and are described in File S1. E protein shows only one canonical mutation (T9I) which is present in all lineages. M protein shows two canonical mutations (Q19E and A63T) that are present in all lineages. Only the BA.5.^ lineage presents an additional mutation (D3N) in the M protein. Mutations R203K and G204R in N protein are common to all lineages whereas mutation P151S has been identified only in BA.4.^.

In this study, we have identified five non-canonical mutations in the M protein (S4F, T7I, L34F, A85V, H125R) and fourteen non-canonical mutations in the N protein (D3V, T49I, H59R, D103N, P162H, N181L, S194L, A220T, G275C, T362I, P365S, T366I, T379I, A414S). Details are provided in Table 4. Several isolates in this study (8%) showed non-canonical mutations in N while a more limited number of isolates showed non-canonical mutations in M (3%). Among the identified Omicron lineages, BA.2.^ and BA.5.^ had the highest percentage of isolates with non-canonical mutations in N (5% and 2%, respectively). Conversely, non-canonical mutations in the gene encoding the M protein were identified exclusively in BA.1.^ and BA.2.^ (2% and 1% of isolates, respectively) (Figure 4). These data are in agreement with previous reports showing a higher frequency of mutations in N protein compared to M in the Omicron variant compared to variants such as Alpha, Gamma, and Delta [51,52,53]. It is of note that N genomic region along with the S gene is widely used in clinical practice to diagnose SARS-CoV-2 positivity [54].

### 3.4. Mutations in Non-Structural Proteins

Mutations identified in non-structural proteins are listed in Table S7 and are described in Supplementary File S1. A total of 115 non-canonical mutations were identified in the ORF1ab gene; 10 non-canonical mutations and 1 deletion were identified in ORF3a; 1 non-canonical mutation was identified in ORF6; 2 non-canonical mutations were identified in ORF7a; 4 non-canonical mutations were identified in ORF8. 

Most non-canonical mutations in ORF1ab were identified in nsp2, nsp3, and nsp1 (18%, 15%, and 13%, respectively), in ORF3a (7% of isolates), in ORF8 (6% of isolates) and in Orf7a (3%). Only one isolate showed a non-canonical mutation in ORF6. 

ORF1ab showed 10 canonical mutations in BA.1.^ and 13 canonical mutations in BA.2.^ and BA.4.^. However, only T3255I and I5967V were common to all Omicron lineages. ORF3a showed only one canonical mutation (T223I) common to BA.2.^, BA.4.^ and BA.5.^. Mutation D61L in ORF6 was present in BA.2.^ and BA.4.^. L11F in ORF7b was present only in BA.4.^. Among the identified Omicron lineages, BA.2.^ and BA.5.^ presented the highest percentage of isolates with non-canonical mutations in ORF1ab (30% and 17%, respectively), and ORF3a (5% and 2%, respectively). 

With respect to other non-structural proteins, no mutations were identified in nsp5, nsp7, nsp8, nsp9, nsp10, or nsp11 while, remarkably, we detected mutations in the catalytic subunit of RdRp polymerase (nsp12) which may have a negative impact on remdesivir efficacy as previously demonstrated [55,56,57]. Within non-structural proteins, nsp1 plays an important role in the evolution of Omicron variants. Nsp1 inhibits host gene expression and suppresses the innate immune response [58,59]. 

We found that nsp1 is one of the genes with the highest frequency of non-canonical mutations. In fact, 27 isolates (13%, N = 27/208) presented 9 non-canonical mutations (V38F, G82C/D, L92V, L107F, V111M, K120N, R124H, N178S) and 3 deletions (82–86 del, 85 del, 141–143 del) in nsp1 (see Table S7). Mutations and deletions of nsp1 were distributed as follows: 50% in BA.2.^ (N = 14), 32% in BA.5.^ (N = 9), and 18% in BA.1.^ (N = 4) and were mutually exclusive. 

In the present study, we also identified three deletions in nsp1: 141–143 del, 82–86 del, and 85 del. Four isolates showed the 141–143 del; eight isolates presented the 82–86 del and five isolates presented the 85 del. Of note, the 141–143 del was described as a canonical alteration of nsp1 in BA.4.^ lineage and as a non-canonical alteration in four isolates of BA.2.^. Deletion 79–89 del is known to be associated with a lower viral load [60]. However, when we compared the Ct values of isolates with or without the deletions in nsp1, we observed that the mean Ct values of isolates carrying the deletions 82–86 del (21.88 ± 3.08, N = 17) or 141–143 del (23.25 ± 3.30, N = 4) were not significantly higher than the Ct values presented by the isolates without deletions (19.98 ± 2.96, N = 185). 

ORF3a protein plays a key role in viral genome replication and virion assembly, inhibition of host mRNA export and translation, and escape from the host immune system [61]. 

We identified 9% of isolates with non-canonical mutations in the gene encoding ORF3a. ORF3a is a viral potassium ion channel (viroporin) that inhibits autophagy and activates the inflammasome and apoptosis [62,63]. We found 11 non-canonical mutations that, to our knowledge, have been poorly characterized so far, except for the L108F and F207L mutations. Although extremely rare, these mutations have been predicted to exert a destabilizing effect on the stability of the protein structure [64]. 

Conversely, ORF8 impairs host cell-mediated immune responses by downregulating MHC-1 molecules [65,66]. ORF8 is poorly conserved among related coronaviruses, likely because of its key role in the pathogenicity of SARS-CoV-2. Many mutations and deletions of ORF8 have been identified [67]. All reports conclude that SARS-CoV-2 can survive without a functional ORF8 though the accuracy of serological tests may be compromised in such cases [68,69]. In this study, we found four non-canonical mutations (Q27*, E64G, A65D, S67F) of the ORF8 protein in 8% of isolates.

## 4. Discussion

The present manuscript provides data regarding the evolution of SARS-CoV-2 spread in Calabria, Southern Italy. 

The first key finding of this study includes the analysis of the diffusion of Omicron variants in Calabria. Omicron spread during 2022 presented a similar trend in Italy and Calabria, with some notable exceptions: (i) recombinant BA.1/BA.2 showed higher frequency in Calabria (13%) than in the rest of Italy (0.02%), and presented all canonical mutations of S protein of BA.2.^ lineage; (ii) BA.2.9, BA.4 and BA.5 emerged in Calabria later than other Italian regions; (iii) BA.1 sub-lineage disappeared in April in Calabria but not in the rest of Italy. 

As observed in this study, the recombinant BA.1/BA.2 increased in Calabria in April (13%) while its frequency was negligible in the rest of Italy (mean frequency 0.02%), with the highest frequency in Friuli Venezia Giulia (0.4%) and the lowest in Lazio (0.02%). 

BA.2.9 occurred for the first time in February in Umbria (0.07%) while in Calabria this sub-lineage was detected for the first time in April. 

BA.4 appeared for the first time in May in Lombardia (0.03%) and BA.5 appeared for the first time in May in Umbria, Lazio, and Puglia (0.09%, 0.04%, and 0.02%, respectively), while the first cases in Calabria were detected in June. 

Finally, unlike what was observed in Calabria where BA.1 disappeared in April, it was still present in Italy until May with cases in Lombardia (0.04%) and Campania (0.002%). In Italy in 2022 COVID-19 showed three peaks of infection associated with the spread of Omicron lineages BA.1, BA.2, BA.4, and BA.5 [38]. In particular, the spread of the BA.1 lineage, from late December 2021 to mid-January 2022, led to an exponential increase in COVID-19 cases, characterized by a 55% increase in the number of diagnosed cases. 

A second peak of infection began in late March with the emergence of the BA.2 lineage. The third peak of infections was registered in June during the transition from the BA.2 lineage to the BA.4 and BA.5 lineages. At the same time, Calabria recorded the same trend of COVID-19 infections as the rest of Italy. 

The second finding of this study was the identification of 10 different sub-lineages of Omicron in Calabria (recombinant BA.1/BA.2, BA.1, BA.1.1, BA.2, BA.2.9, BA.2.10, BA.2.12.1, BA.4, BA.5, BE.1), characterized by the presence of 29–34 canonical mutations. 

The third point was the discovery of 16 non-canonical mutations identified in the S protein as well as 151 non-canonical mutations in the non-structural and accessory proteins of the SARS-CoV-2 genome. The mutations identified in ORF1ab, ORF3a, M, ORF8, and N were not common to all Omicron sub-lineages but showed a specific distribution among variants. 

Non-canonical mutations in the gene encoding S protein occurred preferentially in BA.5 such as, for example, M153I, G181V/A, P209L, A713S, and P862S. Non-canonical mutations in other genes were identified preferentially in BA.2 and BA.5 sub-lineages. For example: BA.2 sub-lineage presented the non-canonical mutations V38F, L107F, G519S, T1760I, G4436V in ORF1ab, L106F and F207L in ORF3a, P365S in N; BA.5 sub-lineage showed the non-canonical mutations H417Y, V559A, T1840I, N4473E, K4483R in ORF1ab, L108F and A54V in ORF3a, T362I in N.

## 5. Conclusions

This study provides a detailed description of the genome evolution of the Omicron variant in the Calabria region of Southern Italy. This analysis allowed the identification of non-canonical mutations in most genes (ORF1ab, S, ORF3a, M, ORF8, N) of the SARS-CoV-2 genome. The non-canonical mutations identified in this study occurred in viral proteins involved in viral replication, assembly and release, viral fusion with cellular membranes, escape from the immune system, resistance to neutralization by antibodies, and resistance to antiviral therapies. The mutations highlighted here show a specific distribution among Omicron sub-lineages allowing discrimination among them. 

In summary, sequencing of the entire SARS-CoV-2 genome allows not only the identification of the exact viral variant with which humans are infected but also real-time monitoring of the emergence of novel mutations that could impact viral transmissibility and the efficacy of therapies.

## Figures and Tables

**Table 1:** Omicron sub-lineages observed in Calabria and in Italy during 2022.

Percentage (%)												
		Recombinant BA.1/BA.2	BA.1	BA.1.1	BA.2	BA.2.9	BA.2.10	BA.2.12.1	BA.4	BA.5	BE.1	Other
January	Calabria	0	78	22	0	0	0	0	0	0	0	0
	Italy	0	66	33	1	0	0	0	0	0	0	0
February	Calabria	0	49	51	0	0	0	0	0	0	0	0
	Italy	0	42	44	11	1	3	0	0	0	0	0
March	Calabria	0	22	49	29	0	0	0	0	0	0	0
	Italy	0	13	22	53	11	1	0	0	0	0	0
April	Calabria	13	0	13	66	6	3	0	0	0	0	0
	Italy	0	2	7	74	16	1	0	0	0	0	0
May	Calabria	0	0	0	45	55	0	0	0	0	0	0
	Italy	0	1	1	78	14	1	1	2	2	0	0
June	Calabria	0	0	0	59	29	0	0	6	6	0	0
	Italy	0	0	0	34	5	0	4	13	37	5	2
July	Calabria	0	0	0	14	2	0	4	9	71	0	0
	Italy	0	0	0	5	1	0	1	11	71	9	2
August	Calabria	0	0	0	0	0	0	0	13	88	0	0
	Italy	0	0	0	1	0	0	0	7	79	7	5
September	Calabria	0	0	0	0	0	0	0	0	94	6	0
	Italy	0	0	0	1	0	0	0	4	78	7	9

**Table 2:** Frequency of non-canonical mutations identified in S protein in Omicron isolates from Calabria during 2022.

Non-Canonical Mutations	Isolates with Non-Canonical Mutations N/368	Isolates with Non-Canonical Mutations %	Omicron Lineages
L5F	9	2.5	BA.2.^; BA.5.^
V6I	1	0.3	BA.2.^
P9T	1	0.3	BA.1.^
S12F	1	0.3	BA.5.^
T22I	1	0.3	BA.1.^
M153I	1	0.3	BA.5.^
D178N	1	0.3	BA.4.^
G181V	1	0.3	BA.5.^
G181A	1	0.3	BA.5.^
P209L	1	0.3	BA.5.^
I624M	1	0.3	BA.2.^
N658S	3	0.8	BA.4.^
A701V	10	2.7	BA.1.^
A713S	2	0.5	BA.5.^
P862S	1	0.3	BA.5.^
E1144V	1	0.3	BA.5.^

^ include all sub-lineages.

**Table 3:** Mutation density observed in the different domains of the S protein detected in isolates from Calabria during 2022. Legend: NTD, N-terminal domain; RBD, receptor binding domain; CT1-CT2, C-terminal 1–C-terminal 2; S1/S2, cleavage site; FP, fusion peptide; HR1, hepta repeat 1; CD1, connector domain 1.

Subdomain of S Protein	Size of Subdomain	Number of Non-Canonical Mutations Observed in Subdomain	Mutation Density (%)
NTD	291 aa	10 (1 in BA.1.^; 2 in BA.2.^; 1 in BA.4.^; 7 in BA.5.^)	3.4
RBD	196 aa	0	0.0
CT1–CT2	157 aa	2 (1 in BA.2.^; 1 in BA.4.^)	1.3
S1/S2 cleavage	129 aa	2 (1 in BA.1.^; 1 in BA.5.^)	1.6
FP	95 aa	1 in BA.5.^	1.1
HR1	65 aa	0	0.0
CD1	76 aa	1 in BA.5.^	1.3

**Table 4:** Mutations identified in genes different from S in Omicron isolates from Calabria during 2022.

Gene	Protein (aa)	Non-Canonical Mutations	Isolates with Non-Canonical Mutations
**ORF1ab**	nsp1 (1–180)	141–143 del	27 (13%)
		82–86 del	
		85 del	
		V38F, G82C/D, L92V, L107F, V111M, K120N, R124H, N178S	
	nsp2 (181–818)	L642F, G392C, S318L, G519S, L624F, G697R, R207C, A566V, A486V, H417Y, S302F, A239V, P361T, P309L, P626L, R545Q, H374Y, A498V, P361S, A690V, T814I, V559A, L204F, A599V, L454F, Q556K, I431M, A801V	37; (18%)
	nsp3 (819–2763)	T1597I, V1211F, S1534I, P1803S, S1612L, T1760I, K1929R, A1473V, L2146F, M1083I, S1188L, H1160Y, A1997V, A1631V, T1496I, G1073V, P1220L, A1809T, T1242I, T1444A, V2116L, Y1465CM, H1545Y, L1450F, D1507N, P2046L, D2037N, A1204T	31; (15%)
	nsp4	S2797F, P2929L, A2784V, W2769R, T2823I, N2272S, A2828V	8; (4%)
	nsp6	I3758V, T3750I, P3767S, L3796F, L3829F	10; (6%)
	nsp12 (4393–5324)	I4498V + E4661N	9; (4%)
		L5141M, H4474Y, G4436V, N4473E, K4483R, I4563M, R4565C, T5131I	
	nsp13 (5325–5925)	S5362P	10; (5%)
		S5360P + D5693G	
		A5703V, S5360P, S5560C, S5398P, M5557I, P5853L, Y5601H	
	nsp14 (5926–6452)	A6532V + V6579F	13; (6%)
		P6128L + G6013S	
		H6208Q + T6564I	
		P6128L, T5941I, G6621R, A6612V, L6082F, L6614F, V6492I, V6107I, V6474L, R6590L	
	nsp15 (6453–6798)	H6789Y	1; (0.5%)
**ORF3a**	ORF3a (1–275)	256–259 del	15; (7%)
		L52F + F207L	
		V13A, A54V, R68G, L106F, L108F, R122I, S171L, E239D	
**M**	Membrane (1–222)	S4F, L34F, T7I, A85V, H125R	6; (3%)
**ORF6**	ORF6 (1–61)	S41C	1; (0.5%)
**ORF7a**	ORF7a (1–121)	L9M, F101fs	2; (1%)
**ORF8**	ORF8 (1–121)	Q27 *, A65D, S67F, E64G	12; (6%)
**N**	Nucleocapsid (1–419)	D3V, T49I, H59R, D103N, P162H, N181L, S194L, A220T, G275C, T362I, P365S, T366I, T379I, A414S	17; (8%)

* stop codon.

## Data Availability

The data presented in this study are openly available in GenBank at accession number SUB12473075.

## Supplementary Materials

The following supporting information can be downloaded at: https://www.mdpi.com/article/10.3390/v15020408/s1. Table S1: Patients’ demographic information and sequencing method; Table S2: Primers list; Table S3: GISAID IDs; Table S4: Omicron distribution in Calabria; Table S5: NGS metrics; Table S6: Omicron canonical mutations; Table S7: Omicron non-canonical mutations found in this study. File S1: Non-canonical mutations details; Figure S1: The distribution of the different sub-lineages in the 4 Calabria areas.
