# Supplementary material for: The Spread of SARS-CoV-2 Omicron Variant in CALABRIA: A Spatio-Temporal Report of Viral Genome Evolution
Source: Viruses. 2023 Jan 31;15(2):408. doi: 10.3390/v15020408 (PMC9963258; doi:10.3390/v15020408)
Supplement: Supplementary file 1 [file viruses-15-00408-s001.zip › File S1.pdf]

Supplemental Material

1. *Non-canonical mutations in non-structural proteins*

1.1 *Nsp2*

Thirty-seven isolates (18%, N=37/208) showed total 28 non-canonical mutations (R207C, A566V, A486V, H417Y, S302F, A239V, P361T, P309L, P626L, R545Q, H374Y, A801V, P361S, A690V, T814I, V559A, L204F, A599V, L454F, Q556K, I431M, A498V, L624F, G697R, G519S, G392C, S318L, L642F) in nsp2 Supplementary Table 7. The non-canonical mutations were distributed among the various sub-lineages as follow: 11% BA.1.^ (N=4); 57% BA.2.^ (N=21); 3% BA.4.^ (N=1); 30% BA.5.^ (N=11) isolates.

1.2 *Nsp3*

Thirty-one isolates (15%, N=31/208) showed total 28 non-canonical mutations (P1803S, S1612L, T1760I, K1929R, A1473V, L2146F, M1083I, S1188L, T1597I, S1534I, V1211F, H1160Y, A1997V, A1631V, T1496I, G1073V, P1220L, A1809T, T1242I, T1444A, V2116L, Y1465CM, H1545Y, L1450F, D1507N, P2046L, D2037N, A1204T) in nsp3 Supplementary Table 7. These Non-canonical mutations were distributed among the various sub-lineages as follow: isolates BA.1.^ (N=5, 16%); BA.2.^ (N=19 61%); BA.4.^ (N=2 7%); BA.5.^ (N=5 16%).

1.4 *Nsp4*

Eight isolates (4%, N=8/208) showed total 7 non-canonical mutations (S2797F, P2929L, A2784V, W2769R, T2823I, N2272S, A2828V) in nsp4 Supplementary Table 7. Non-canonical mutations described before were distributed among the various sublineages as follow: isolates BA.2 (N=4 50%); N=4.^ BA.5 (50%).

1.5 *Nsp6*

Eleven isolates (6%, N=10/172) showed total 5 non-canonical mutations (I3758V, T3750I, P3767S, L3796F, L3829F) in nsp6 Supplementary Table 7. Non-canonical mutations described before were distributed among the various sublineages as follow: isolates BA.1. ^ (N=1, 10%); BA.2.^ (N=9 90%).

1.6 *Nsp12-13-14-15*

Thirty-three isolates showed non-canonical mutations in nsp12-13-14-15 (16%, N=33/208) Supplementary Table 7. Overall 35 non-canonical mutations were distributed as follows: N=10

(I4498V, E4661N, L5141M, H4474Y, G4436V, N4473E, K4483R, I4563M, R4565C, T5131I) in nsp12 , N=9 (S5360P, A5703V, S5560C, S5398P, S5362P, D5693G, M5557I, P5853L, Y5601H) in nsp13, N=15 (T5941I, G6621R, A6612V, A6532V, V6579F, L6082F, P6128L, G6013S, H6208Q, T6564I, L6614F, V6492I, V6107I ,V6474L, R6590L) in nsp14, N=1 (H6789Y) in nsp15. Non-canonical mutations described before were distributed among the various sub-lineages as follow: isolates BA.1.^ (N=4 11%); BA.2.^ (N=20 57%); BA.4.^ (N=1 3%); BA.5.^ (N=10 29%).

1.7 *ORF3a*

Fifteen isolates (7%, N=15/208) showed total 10 non-canonical mutations (V13A, L52F, A54V, R68G, L106F, L108F, R122I, S171L, F207L, E239D) and one deletion (DelV256/V259) in ORF3a protein Supplementary Table 7. Non-canonical mutations described before were distributed among the various sublineages as follow: isolates BA.1. ^ (N=1, 7%), BA.2.^ (N=10, 66%); BA.5.^ (N=4 27%).

1.8 *ORF6a*

In one isolate of BA.2 sub-lineage we identified mutation S41C in ORF6.

1.9 *ORF7a*

In two isolates of BA.2. ^ showed the following non-canonical mutations in ORF7A (L9M and F101fs).

1.10 *ORF8*

Twelve isolates (6%, N=12/208) showed total 4 non-canonical mutations (Q27*, E64G, A65D, S67F) in ORF8 protein Supplementary Table 7. Non-canonical mutations described before were distributed among the various sub-lineages as follow: isolates BA.2.^ (N=8 67%); N=3 in BA.2.9 (25%); BA.5.^ (N=4 33%).

2. *Non-canonical mutations in structural proteins*

2.1 *M protein*

Six isolates (3%, N=6/208) showed total 5 non-canonical mutations (SF4, T7I, L34F, A85V, H125R) in M protein Supplementary Table 7. Non-canonical mutations described before were distributed among the various sublineages as follow: isolates BA.1. ^ (N=4 67%); BA.2.^ (N=2 33%);

2.2 *N protein*

Seventeen isolates (8%, N=17/208) showed total 14 non-canonical mutations (D3V, T49I, H59R, D103N, P162H, N181L, S194L, A220T, G275C, T362I, P365S, T366I , T379I, A414S) in N protein Supplementary Table 7. Non-canonical mutations described before were distributed among the various sub-lineages as follow: BA.2. ^ (N=11 65%); BA.4.^ (N=1 6%); BA.5.^ (N=5 29%).
