# Supplementary figures and images for: The Spread of SARS-CoV-2 Omicron Variant in CALABRIA: A Spatio-Temporal Report of Viral Genome Evolution
Source: Viruses. 2023 Jan 31;15(2):408. doi: 10.3390/v15020408 (PMC9963258; doi:10.3390/v15020408)

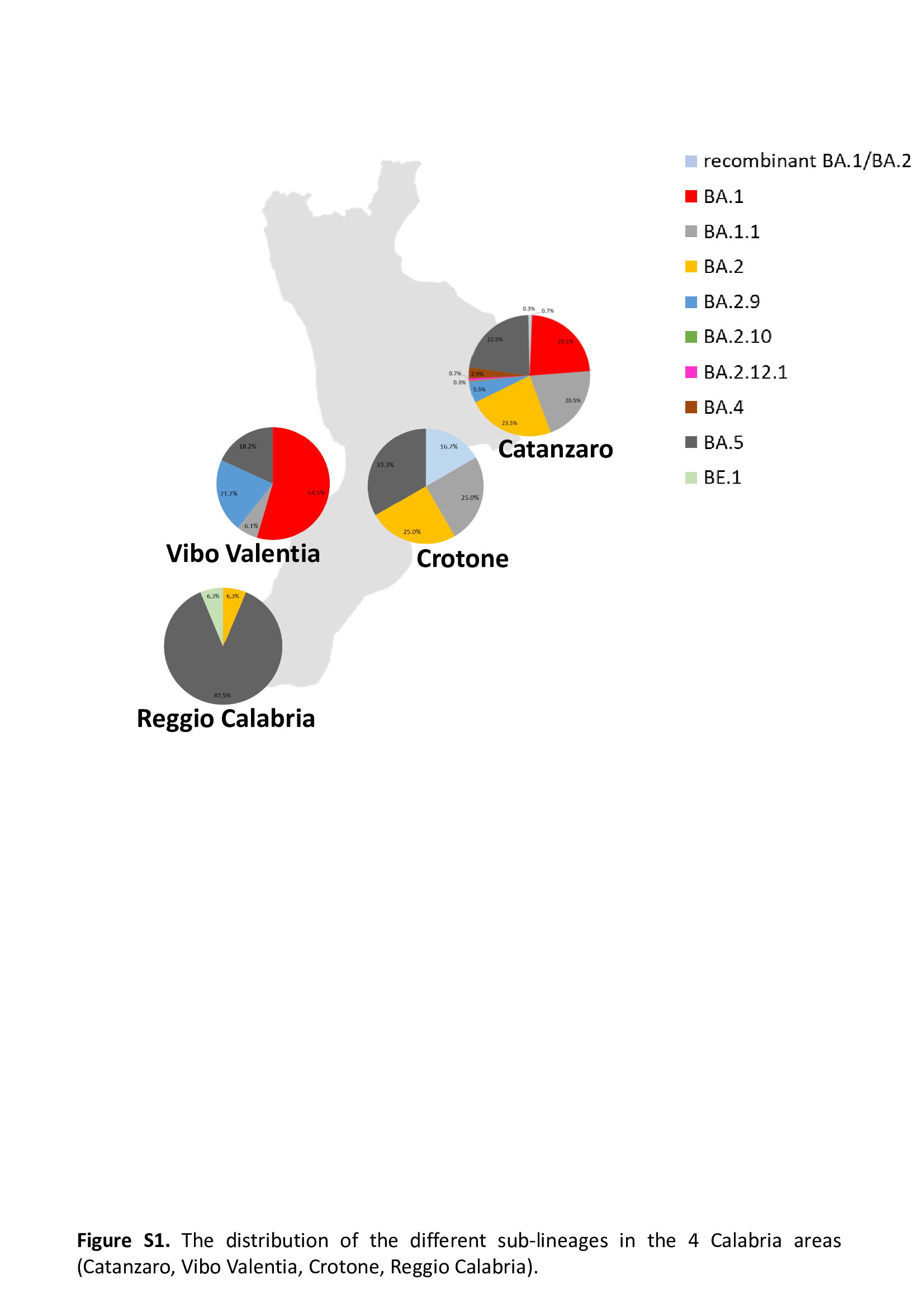

Supplement: Supplementary file 1 [file viruses-15-00408-s001.zip › Figure S1.tiff]
